# Is serum PSA a predictor of male hypogonadism? Testing the hypothesis

**DOI:** 10.20945/2359-3997000000326

**Published:** 2021-02-15

**Authors:** Collins Amadi, Kinikanwo I. Green, Ehimen P. Odum

**Affiliations:** 1 Rivers State University Department of Chemical Pathology Port Harcourt Rivers State Nigeria Department of Chemical Pathology, Rivers State University, Port Harcourt, Rivers State, Nigeria.; 2 University of Port Harcourt Teaching Hospital Department of Obstetrics and Gynecology Port Harcourt Rivers State Nigeria Department of Obstetrics and Gynecology, University of Port Harcourt Teaching Hospital, Port Harcourt, Rivers State, Nigeria.; 3 University of Port Harcourt Teaching Hospital Department of Chemical Pathology Port Harcourt Rivers State Nigeria Department of Chemical Pathology, University of Port Harcourt Teaching Hospital, Port Harcourt, Rivers State, Nigeria.

**Keywords:** Male hypogonadism, total PSA, free PSA

## Abstract

**Objective:**

Male hypogonadism (MH) is common among infertile men. Besides testosterone, limited MH biomarkers are available, while researchers have suggested the use of prostate-specific antigen (PSA) to help diagnose MH. Hence, we sought to evaluate the potential use of PSA to predict MH among relatively young men with infertility in Nigeria.

**Materials and methods:**

The study included 707 male partners (35–44 years) in infertile couples seeking infertility evaluation at a third-level care center in Nigeria. MH was diagnosed using standard guidelines. Receiver operating characteristic (ROC) and regression analyses explored the potential of serum free PSA (fPSA) and total PSA (tPSA) in predicting MH and MH-related clinical features.

**Results:**

In all, 29.7% of the patients had MH (MH+ve). The MH+ve group had lower mean values of fPSA and tPSA than the group without MH (MH-ve). The best fPSA threshold of < 0.25 μg/L compared with the best tPSA threshold of < 0.74 μg/L had higher accuracy (area under the curve [AUC] 0.908 versus 0.866, respectively), sensitivity (87% versus 83%, respectively), and specificity (42% versus 37%, respectively) for MH diagnosis. After adjustment for confounders, fPSA level ≤ 0.25 μg/L was more likely to predict MH-related decreased libido (odds ratio [OR] 2.728, p<0.001) and erectile dysfunction (OR 3.925, p<0.001) compared with tPSA ≤ 0.74 μg/L in the MH+ve group.

**Conclusion:**

For MH diagnosis, fPSA and tPSA had good sensitivity but very poor specificity, although fPSA had better potential for MH diagnosis and association with MH-related clinical features than tPSA. Hence, fPSA could complement other biomarkers for MH diagnosis in men 35–44 years, although we recommend further studies to confirm these findings.

## INTRODUCTION

Male hypogonadism (MH) is a spectrum of clinical syndromes associated with diminished testicular function due to testosterone deficiency or impaired action (1,2). Characteristic findings of MH include semen abnormalities, sexual dysfunction, infertility, metabolic changes, and psychosocial abnormalities (2–4). The etiology of MH may be primary, secondary, congenital, or acquired (1–3). The occurrence of MH is common among men presenting with infertility (2,5). In northern Nigeria, a 45.9% prevalence of MH has been documented among men seeking infertility evaluation (2,5). Evidence indicates that MH is associated with various other noncommunicable disorders among men (6).

MH is a major endocrine disorder in men, regardless of race, socioeconomic status, or geographical location (4–6). Men of all ages can be affected, and the clinical presentation depends on the timing of puberty onset (2–5). Testosterone synthesis tends to decline after the age of 40 years at a yearly rate of 0.4–2% in men (1,2). This decline has been linked to a predominance of MH in older individuals (3–6). Even though MH is common among elderly individuals, epidemiologic evidence also indicates it as having a high incidence in younger men (5–7), although the clinical manifestations of MH are less pronounced in these individuals compared with older men (7).

The parameter most widely used for MH diagnosis remains the accurate laboratory determination of decreased serum testosterone levels with concomitant MH clinical features (4). Other biomarkers have been suggested, but they have yet to gain popularity in clinical practice (8). Serum testosterone determination is highly subject to various preanalytical and biological factors, especially among older individuals. These factors have resulted in a search for other biomarkers to help establish the diagnosis of MH (8,9).

Testosterone exerts multisystemic functions in men (10). The most pronounced effect is on the prostate, where testosterone influences the expression of the prostate-specific antigen (*PSA*) gene initiating PSA synthesis (11). The influence of testosterone on PSA synthesis has prompted the interest of some investigators suggesting that PSA is a biomarker of MH (12). However, most of these investigators had evaluated PSA levels mainly among middle-aged and older men, who are likely to have prostate disorders and other comorbid conditions that could affect the association between MH and PSA (12–14).

The association between PSA and testosterone is less variable in younger men, and a positive correlation between PSA and testosterone levels has been consistently established in younger compared with older men (13). Additionally, the “saturation” theory suggests that the relationship is mainly evident at low testosterone concentration, which may favor MH diagnosis (15).

Hence, the present study aimed to evaluate the predictive potentials of serum total PSA (tPSA) and free PSA (fPSA) on MH and MH-related features among young men of Nigerian origin presenting for infertility evaluation.

## MATERIALS AND METHODS

### Study location and design

This prospective, descriptive, and cross-sectional survey was conducted at the University of Port Harcourt Teaching Hospital (UPTH) in Nigeria. UPTH is a third-level health care facility located along the famous East-West Road in Nigeria. The hospital is a major referral center in the southern part of the country and has various specialized departments.

### Ethical considerations

The study protocol was approved by the Institutional Research Ethics Committee (approval reference UPTH/ADM/90/S.11/VOL.XI/851). Informed consent was submitted by all subjects upon enrollment. The study was conducted according to the UPTH research ethics standards and the Declaration of Helsinki.

### Sample size determination and sampling protocol

The minimum sample size required for the study was 377, which was computed using the formula $\text{n}={\text{Z}}^{2}\text{pq}/{\text{d}}^{2}$ (n = sample size, Z = standard normal deviation – set at 1.96, p = proportion in the target population estimated to have the particular characteristics of interest [MH]), using 45.9% as reported within the study region, q = 1 – p (proportion in the target population not having the characteristics of interest, d = degree of accuracy required, set at 0.05) with a 95% confidence interval (CI) and a power of 80% (5,16). To enhance the study power, we recruited 707 individuals.

Convenience sampling was applied to recruit the participants from March 2018 to January 2020.

### Study population

The study population comprised male partners in infertile couples recruited from the Departments of Obstetrics-Gynecology and Chemical Pathology outpatient units. In these units, male partners in infertile couples usually undergo endocrine evaluation following abnormal seminogram findings.

### Eligibility criteria

The criteria for inclusion were (1) male partners in infertile couples with abnormal seminogram findings, (2) age between 35–44 years, (3) tPSA < 1.5 μg/L, and (4) International Prostate Symptom Score (IPPIS) ≤ 1 (17).

The criteria for exclusion were (1) age outside 35–44 years, (2) tPSA > 1.5 μg/L, (3) abnormal (enlargement or nodularity) digital rectal examination (DRE) findings, (4) IPPIS score ≥ 2, (5) abnormal transrectal ultrasound scan (TRUS) of the prostate gland, (5) previous or existing history of prostatitis, any malignant conditions, genetic disorders of the hypothalamus-pituitary-gonadal axis, diabetes mellitus, thyroid disorders, acromegaly, hyperprolactinemia, hepatic disorders, chronic renal diseases, chronic obstructive pulmonary disease, autoimmune disorders, human immunodeficiency virus (HIV) disease, major anatomical disorders or surgery of the urological system, major psychiatric disorders, alcohol or illicit drug abuse, and use of exogenous androgens/glucocorticoid/opioid/anticonvulsants/progestins/estrogens.

Other criteria for exclusion were lack of consent, presence of any chronic debilitating illness, prior prostatectomy and orchiectomy, and use of medications known to influence serum PSA levels such as statins, nonsteroidal antiinflammatory agents, thiazide, calcium supplement, aspirin, 5-alpha-reductase inhibitors, alpha-blockers, other anti-androgens, and exogenous testosterone.

### Acquisition of non-laboratory data

Data on clinical and anthropometric variables obtained included age, systolic blood pressure (SBP, mmHg), diastolic blood pressures (DBP, mmHg), weight (kg), height (m), body mass index (BMI) calculated with the formula weight (kg)/height (m^2^), and waist circumference (cm). Prostate volume (cm^3^) was calculated using the prolate ellipsoid formula with dimensions obtained from TRUS of the prostate (18). The IPPIS and Androgen Deficiency in the Aging Male (ADAM) questionnaires were also administered (17,19).

While the IPPIS questionnaire evaluated urinary symptoms over the preceding 4 weeks due to prostate enlargement (with 0–7 denoting no or mild symptoms), the ADAM questionnaire was a validated screening tool for identifying men at risk of MH (17,19).

The ADAM questionnaire consisted of 10 questions with “yes” or “no” answers concerning MH symptoms. A positive ADAM questionnaire response is a “yes” answer to either question 1 (reduced libido) or 7 (erectile dysfunction) or any three other questions (19).

### Acquisition of laboratory data

Seminogram was carried out in the center using standard protocols (20). The parameters determined were sperm count (normal >15 x 10^9^/mL), sperm morphology (normal > 4%), sperm total motility (normal > 40%), and semen volume (normal 1.5 mL), as defined by the World Health Organization (WHO) (20). Identified cases with abnormal seminogram results were usually confirmed on repeat analysis within 2–4 weeks.

A total of 8 mL of 10–12-hour fasting venous blood was drawn between 8–10 am from each participant's antecubital vein. Of that, 5 mL were added to tubes without anticoagulant and allowed to clot undisturbed for 1 hour for full retraction, then centrifuged at 2500 revolutions per minute. The serum supernatant was separated with a Pasteur's pipette into tubes without anticoagulant and frozen at −80°C until analysis of tPSA, fPSA, serum follicle-stimulating hormone (sFSH), serum luteinizing hormone (sLH), serum prolactin (sPRL), and serum total testosterone (sTT). The remaining 3 mL of blood drawn were added to an ethylene diamine tetra-acetic acid tube and centrifuged immediately. The supernatant plasma was separated and stored frozen until analysis of plasma sex–hormone-binding globulin (SHBG) and albumin.

Plasma tPSA, fPSA, sFSH, sLH, sPRL, sTT, and SHBG were measured using enzyme-linked immunosorbent assay method with standard reagents (Monobind Incorporated, Lake Forest, CA, USA). Plasma albumin was determined using the bromocresol green colorimetric method (Randox Laboratories Ltd, Crumlin, United Kingdom).

Free testosterone (cFT) and bioavailable testosterone (cBioT) were estimated using online calculators (http://www.issam.ch/freetesto.htm) according to Vermeulen's formula (21).

Following analysis, samples with sTT in the hypogonadal range were identified, and the patient was contacted for repeat sampling and laboratory analysis within 2–4 weeks for confirmation, based on Endocrine Society guidelines (4).

Quality control specimens (Monobind Incorporated) with known hormone concentrations spanning higher and lower concentrations were used to determine analytical precision.

### Data Definition and Stratifications

A) MH+ve (men positive for hypogonadism): Strictly defined as those men meeting all of the following four conditions:

- sTT ≤ 8.0 nmol/L (based on the joint clinical practice guideline of the International Society of Andrology, International Society for the Study of Aging Male, and the European Urology Association, which define Androgen Deficiency as sTT ≤ 8.0 nmol/L) in two or more samples collected at different times (22).
- fT ≤ 0.18 nmol/L in two or more samples collected at different days, as defined by the Endocrine Society (23).
- Positive response to at least any one of the sexual domain items in the ADAM questionnaire (loss of libido and erectile dysfunction) (19).
- Low sperm count (< 15 x 10^9^/mL) with at least one other abnormal seminogram parameter on at least two separate occasions.

B) MH-ve (men negative for hypogonadism): Those men who did not meet the above four listed criteria.

### Data management and statistical analysis

The acquired data was entered into the software SPSS (version 21.0; SPSS Inc. Chicago, IL, USA) and subsequently analyzed. Continuous data were expressed as mean ± standard deviations and compared with independent Student's *t* test. Categorical data were expressed using numbers and percentages and compared with the chi-square test. The diagnostic potentials and the optimal cutoff values of tPSA and fPSA to predict MH were determined using receiver operating characteristics (ROC) curve analysis. Binary logistics regression analysis was used to evaluate relationships between variables. Statistical differences were considered significant at p *<* 0.05.

## RESULTS

During the study period, 2,820 infertile couples presented for infertility evaluation in the study center. Applying the eligibility criteria, a total of 768 male partners in infertile couples met the eligibility criteria and were enrolled. However, 61 were further excluded due to failure to present for blood or semen sampling (n = 21), inability to present for repeat blood sampling (n = 15), inability to present for repeat seminogram (n = 15), and abnormal DRE and TRUS finding (n = 10), yielding a final population sample of 707 patients. Among these, MH+ve was documented in 210 (29.7%) patients, while 497 (70.3%) were categorized as MH-ve.

Most MH+ve patients (60.5%, n = 127) were older than 40 years and were predominantly (53.3%, n = 112) categorized into the secondary infertility class. The majority of the MH+ve patients responded positively (“yes” responses) to the questions about decreased libido (80%, n = 168, p < 0.001) and erectile dysfunction (90%, n = 189, p < 0.001) (Table 1).

**Table 1:** Comparison of categorical variables between study cohorts

Variable		Entire cohort n = 707	MH+ve n = 210	MH-ve n = 497	P value
Age, n (%)					
	≤ 40 yrs	408 (57.7)	83 (39.5)	325 (65.4)	<0.001*
	>40 yrs	299 (42.3)	127 (60.5)	172 (34.6)	
Infertility class, n (%)					
	Primary	343 (48.5)	98 (46.7)	245 (49.3)	<0.001*
	Secondary	364 (51.5)	112 (53.3)	252 (50.7)	
ADAM item 1 (Decreased libido), n (%)					
	Yes	302 (42.6)	168 (80)	133 (26.8)	<0.001*
	No	406 (57.4)	42 (20)	364 (73.2)	
ADAM item 7 (Erectile dysfunction), n (%)					
	Yes	252 (35.6)	189 (90)	63 (12.7)	< 0.001*
	No	455 (64.4)	21 (10)	434 (87.3)	

* Statistically significant;

MH+ve: men positive for hypogonadism; MH-ve: men negative for hypogonadism.

Participants in the MH+ve group had higher mean age and SBP, DBP, weight, BMI, WC, FSH, LH, prolactin, and SHBG values (p < 0.05), and lower mean levels of albumin, TT, cFT, cBioT, tPSA, fPSA, prostate volume, sperm count, sperm motility, sperm morphology, and semen volume (p < 0.05) (Table 2).

**Table 2:** Comparison of noncategorical variables between study cohorts

Variable	Entire cohort, n = 707 (Mean ± SD)	MH+ve, n = 210 Mean ± SD)	MH-ve, n = 497 (Mean ± SD)	P value
Age, years	39.89 ± 2.80	41.13 ± 2.82	39.38 ± 2.63	< 0.001*
SBP, mmHg	128.32 ± 6.67	129.33 ± 8.20	127.80 ± 6.00	0.009*
DBP, mmHg	76.53 ± 6.02	78.33 ± 6.30	75.80 ± 5.73	< 0.001*
Weight, kg	82.70 ± 7.18	84.68 ± 6.38	81.56 ± 7.20	0.006*
Height, m	1.71 ± 0.12	1.71 ± 0.14	1.71 ± 0.12	0.775
BMI, kg/m^2^	28.34 ± 3.01	29.32 ± 2.89	27.92 ± 2.94	< 0.001*
WC, cm	96.11 ± 6.01	97.30 ± 6.74	97.03 ± 5.76	< 0.001*
Prostate volume, cm^3^	25.54 ± 3.63	21.40 ± 2.13	27.30 ± 3.10	< 0.001*
Serum FSH, IU/L	8.90 ± 3.73	11.42 ± 3.82	7.85 ± 3.14	< 0.001*
Serum LH, IU/L	6.47 ± 2.50	9.08 ± 2.28	5.37 ± 1.82	< 0.001*
Serum prolactin, μg/L	8.30 ± 2.40	8.48 ± 2.41	8.21 ± 2.30	0.461
Plasma SHBG, mmol/L	24.02 ± 3.76	43.21 ± 3.51	15.90 ± 2.44	< 0.001*
Plasma albumin, g/L	34.34 ± 4.30	33.70 ± 3.7	36.07 ± 2.80	< 0.001*
Serum TT, nmol/L	15.21 ± 4.33	5.68 ± 1.11	19.23 ± 3.21	< 0.001*
Serum cFT, nmol/L	0.53 ± 0.38	0.14 ± 0.10	0.70 ± 0.21	< 0.001*
Serum cBioT, nmol/L	2.92 ± 1.38	1.51 ± 0.51	3.50 ± 1.90	< 0.001*
Serum tPSA, μg/L	0.83 ± 0.32	0.53 ± 0.30	0.96 ± 0.23	< 0.001*
Serum fPSA, μg/L	0.27 ± 0.13	0.14 ± 0.11	0.34 ± 0.08	< 0.001*
Sperm count, per 10^9^/L	11.64 ± 2.51	10.21 ± 2.03	12.28 ± 2.51	< 0.001*
Sperm motility, %	28.45 ± 2.14	22.63 ± 4.84	30.90 ± 2.60	< 0.001*
Sperm morphology, %	5.94 ± 3.72	4.07 ± 2.25	6.73 ± 3.60	< 0.001*
Semen volume, mL	1.62 ± 0.38	1.40 ± 0.30	1.70 ± 0.33	< 0.001*

* Statistically significant;

MH+ve: men positive for hypogonadism; MH-ve: men negative for hypogonadism; mmHg: millimeter of mercury; kg: kilogram; m; meters; cm: centimeters; μg/L: microgram per liter; mmol/L: millimole per liter; nmol/L: nanomole per liter; SD: standard deviation; SBP: systolic blood pressure; DBP: diastolic blood pressure; BMI: body mass index; WC: waist circumference; FSH: follicle-stimulating hormone; LH: luteinizing hormone; SHBG: sex–hormone-binding globulin; TT: total testosterone; cFT: calculated free testosterone; cBioT: calculated bioavailable testosterone; tPSA: total prostate-specific antigen; fPSA: free prostate-specific antigen.

As shown in Table 3, tPSA had a ROC area under the curve (AUC) value of 0.866 with an optimal diagnostic cutoff value of 0.74 μg/L yielding 83% sensitivity and 37% specificity for MH+ve diagnosis. However, fPSA had a more perfect accuracy (ROC AUC 0.908), suggesting a higher diagnostic potential for MH+ve than tPSA, with an optimal diagnostic cutoff value of fPSA at 0.25 μg/L rendering 87% sensitivity and 42% specificity for MH +ve diagnosis.

**Table 3:** Results of receiver operating characteristics (ROC) curve analysis of the performance of serum total prostate-specific antigen (tPSA) and free prostate-specific antigen (fPSA) in the diagnosis of hypogonadism (MH+ve)

	AUC	95% CI	P value	Optimal cutoff value for MH+ve diagnosis	Cutoff value sensitivity	Cutoff value specificity
tPSA	0.866	0.840 − 0.892	< 0.001*	0.74 μg/L	83%	37%
fPSA	0.908	0.884 − 0.931	< 0.001*	0.25 μg/L	87%	42%

* Statistically significant;

MH+ve: men positive for hypogonadism; μg/L: microgram per liter; AUC: area under the curve; CI: confidence interval.

Table 4 shows that a tPSA < 0.74 μg/L predicted decreased libido (odds ratio [OR] 1.254, 95% CI 0.598–2.966, p < 0.001) and erectile dysfunction (OR 1.856, 95% CI 0.169–2.172, p < 0.001) in the MH+ve group (Table 3, Panel A). In the adjusted regression analysis, a fPSA < 0.25 μg/L was more likely to predict decreased libido (OR 2.728, 95% CI 1.349–4.721, p < 0.001) and erectile dysfunction (OR 3.925, 95% CI 1.225–5.984, p < 0.001) compared with a tPSA < 0.74 μg/L in the MH+ve group (Table 3, Panel B).

**Table 4:** Associations between total prostate-specific antigen (tPSA) and free prostate-specific antigen (fPSA) with sexual domain items of the Androgen Deficiency in the Aging Male (ADAM) questionnaire in the group of men positive for hypogonadism (MH+ve)

Panel A. tPSA (using cutoff value ≤ 0.74 μg/L)				
**ADAM test item 1**	**Decreased libido?**	**OR** †	**95% CI**	**P value**
	No	Reference	–––	
	Yes	1.254	0.598−2.966	< 0.001*
**ADAM test item 7**	**Erectile dysfunction?**			
	No	Reference	–––	
	Yes	1.856	0.169−2.172	< 0.001*

Panel B. fPSA (using a cutoff value ≤ 0.25 μg/L)				
**ADAM test item 1**	**Decreased libido?**	**OR** †	**95% CI**	**P value**
	No	Reference	–––	< 0.001*
	Yes	2.728	1.349−4.721	
**ADAM test item 7**	**Erectile dysfunction?**			
	No	Reference	––––	
	Yes	3.925	1.225−5.984	< 0.001*

† Adjusted for age, body mass index (BMI), prostate volume, systolic blood pressure (SDP), diastolic blood pressure (DBP), waist circumference (WC), sex−hormone-binding globulin (SGBG), plasma albumin, follicle-stimulating hormone (FSH), luteinizing hormone (LH), serum total testosterone (sTT), tPSA, sperm count, sperm motility, sperm morphology, and sperm volume;

* statistically significant; tPSA: total prostate-specific antigen; fPSA: free prostate-specific antigen; CI: confidence interval; μg/L: microgram per liter.

## DISCUSSION

The occurrence of MH is common among infertile patients (5,24). Geidem and cols. evaluated the hormonal profile of men investigated for infertility in northern Nigeria and reported a 45.9% prevalence of MH in their study cohort (5). Although this prevalence rate is higher than the one documented in the present study (29.7%), this could be due to differences in study designs. We studied men aged 35–44 years and used a set of criteria for MH diagnosis, while Geidem and cols. evaluated men aged 22–52 years using only a single criterion for MH diagnosis. Other higher and lower variations in MH frequency have been documented in the literature (24).

Apart from sTT, there are limited numbers of biomarkers described in the literature to predict MH accurately (8). Some other biomarkers, such as insulin-like factor 3, have been suggested and evaluated but have yet to gain popularity in clinical practice (25). The *PSA* gene is among the genes positively influenced by sTT (11). Among men with suboptimal sTT status, a positive correlation between PSA and sTT has been documented (13,15). At optimal sTT status, the androgen receptor is saturated, and further rises in sPSA in normal sTT status are independent of the sTT influence (15). This relationship has prompted few studies geared towards the evaluation of sPSA properties over MH diagnosis (12,13).

We evaluated the diagnostic properties of tPSA and fPSA over MH among relatively young men and demonstrated that both molecular PSA forms predict MH+ve significantly with good sensitivity but very poor specificity, thereby making both molecules poor diagnostic parameters for MH+ve diagnosis among young men. Compared with the strict diagnostic criteria for MH diagnosis applied in the present study, tPSA showed good diagnostic properties in predicting MH+ve, although better properties were established by fPSA.

At the best sensitivity and specificity to perfectly predict MH by fPSA, obtained at < 0.25 μg/L, this marker demonstrated better sensitivity, specificity, PPV, and NPP than that rendered by the best tPSA cutoff threshold of < 0.74 μg/L. Our findings are in line with a closely similar diagnostic study by Rastrelli and cols., who evaluated the diagnostic properties of tPSA (but not of fPSA) on MH diagnosis and documented observations, although with slightly different diagnostic properties than ours (12).

Compared with the diagnostic properties of tPSA demonstrated in the present study, Rastrelli and cols. reported lower accuracy (AUC 0.612 ± 0.001) and sensitivity (65.2%) but higher specificity (55.5%) over MH diagnosis at best tPSA threshold of < 0.65 μg/L (12). However, in contrast to our study design, Rastrelli and cols. evaluated predominantly middle-aged and older individuals, which may have contributed to the variations in diagnostic properties obtained in their study and ours.

When the MH-related sexual domains (erectile dysfunction and decreased libido) of the ADAM questionnaire were evaluated in the present study, a fPSA threshold of ≤ 0.25 μg/L also exhibited a greater likelihood of predicting erectile dysfunction and decreased libido in the MH+ve group compared with a tPSA threshold of ≤ 0.74 μg/L. Our findings compare with those of other studies in the literature reporting low tPSA association with MH-related sexual dysfunction (SD) (12,13).

In a retrospective cross-sectional study of patients presenting with SD, Coronal and cols. observed that subjects with higher tPSA levels experienced lower frequencies of SD (including erectile dysfunction and loss of libido) (13). Rastrelli and cols. had also concluded in their study that low tPSA was associated with impaired sex- and sleep-related erections (12).

The relatively better diagnostic properties rendered by the fPSA over MH diagnosis could be attributed to some factors (26,27). According to some data, fPSA – and not tPSA or age – has a better predictive value of prostate volume, which ultimately determines PSA secretion and serum concentration (24). Levels of fPSA have been documented to be highly sensitive to slight changes in sTT compared with those of tPSA (15,26,27).

The “saturation” hypothesis proposes that PSA is sensitive to TT variations at or below the near-severe hypogonadism level (8 nmol/L, the level applied in the present study) but is insensitive to testosterone variations above this concentration (15). Hence, we hypothesize that the changes inherent in PSA, as proposed by the “saturation” hypothesis, may be more pronounced with fPSA since this is the physiologically active molecular fraction of PSA instead of tPSA.

The study has some limitations worthy of note. First, it was a single–hospital-based survey, and the findings may not represent the entire population within the study region. Second, the study's findings were obtained from men of black race, which may also not reflect those of Caucasian populations. Third, we relied on calculated serum testosterone parameters, due to limited resources, instead of directly measuring these parameters, although the correlation between calculated and directly measured testosterone parameters has already been established (21). Fourth, being a cross-sectional study, the findings do not allow deduction of a cause-and-effect relationship, and the duration of the study does not guarantee stability to the associations observed between variables over different time periods.

In conclusion, MH is common among infertile couples and has limited biomarkers dedicated to its diagnosis. Investigators have previously suggested the usefulness of tPSA in identifying MH, which prompted this study. Although both fPSA and tPSA showed good sensitivity for MH diagnosis, they also had very poor specificity, making both molecules poor diagnostic parameters for MH evaluation in young men. Still, fPSA had better MH diagnostic potential and association with MH-related clinical features than tPSA. Hence, fPSA could complement other biomarkers for MH diagnosis among relatively young men. Further studies are suggested to confirm the findings of the present study.

## References

[1] McBride JA, Carson CC, Coward RM (2016). Testosterone deficiency in the aging male. Ther Adv Urol.

[2] Lo EM, Rodriguez KM, Pastuszak AW, Khera M (2018). Alternatives to Testosterone Therapy: A Review. Sex Med Rev.

[3] Ohlander SJ, Lindgren MC, Lipshultz LI (2016). Testosterone and male infertility. Urol Clin North Am.

[4] Bhasin S, Cunningham GR, Hayes FJ, Matsumoto AM, Snyder PJ (2010). Testosterone therapy in men with androgen deficiency syndromes: an endocrine society clinical practice guideline. J Clin Endocrinol Metab.

[5] Geidam AD, Yawe KDT, Adebayo AEA, Idrisa A (2008). Hormonal profile of men investigated for infertility at the University of Maiduguri in northern Nigeria. Singapore Med J.

[6] Modhu SV (2017). Hypogonadism and diabetes mellitus – Implications for cardiovascular risk. In J Diabetes Dev Countries.

[7] Tajar A, Huhtaniemi IT, O’Neill TW, Finn JD, Pye SR, Lee DM (2012). Characteristics of androgen deficiency in late-onset hypogonadism: results from the European Male Aging Study (EMAS). J Clin Endocrinol Metab.

[8] Karakas SE, Surampudi P (2018). New biomarkers to evaluate hyperandrogenic women and hypogonadal men. Adv Clin Chem.

[9] Trost LW, Mulhall JP (2016). Challenges in testosterone measurement, data Interpretation, and methodological appraisal of interventional trials. J Sex Med.

[10] Bain J (2007). The many faces of testosterone. Clin Interv Aging.

[11] Banerjee PP, Banerjee S, Brown TR, Zirkin BR (2018). Androgen action in prostate function and disease. Am J Clin Exp Urol.

[12] Rastrelli G, Corona G, Vignozzi L, Masenli E, Silveri A, Monami M (2013). Serum PSA as a predictor of testosterone deficiency. J Sex Med.

[13] Corona G, Boddi V, Lotti F, Gacci M, Carini M, De vita G (2010). The relationship between testosterone to prostate-specific antigen in men with sexual dysfunctions. J Sex Med.

[14] Wittert GA, Chapman IM, Haren MT, Mackintosh S, Coates P, Morley JE (2002). Oral Testosterone Supplementation Increases Muscle and Decreases Fat Mass in Healthy Elderly Males With Low–Normal Gonadal Status. J Gerontol.

[15] Morgentaler A (2013). Testosterone therapy in men with prostate cancer: scientific and ethical considerations. J Urol.

[16] Naing L, Winn T, Rush BN (2006). Practical issues in calculating sample size for prevalence studies. Arch Orofacial Sci.

[17] Barry MJ (1992). The American Urological Association symptom index for benign prostatic hyperplasia. J Urol.

[18] Lee JS, Chung BH (2007). Trans-rectal ultrasound versus magnetic resonance imaging in the estimation of prostate volume as compared with radical prostatectomy specimens. Urol Int.

[19] Morley JE, Charlton E, Patrick P, Kaiser FE, Cadeau P, McCready D (2000). Validation of a screening questionnaire for androgen deficiency in aging males. Metabolism.

[20] World Health Organization (2010). WHO Laboratory Manual for the Examination and Processing of Human Semen.

[21] Vermuelen A, Verdonck L, Kaufman JM (1999). A critical evaluation of simple methods for the estimation of free testosterone in serum. J Clin Endocrinol Metab.

[22] Nieschlag E, Swerdloff R, Behre HM, Gooren LJ, Kaufman JM, Legros JJ (2008). Investigation, treatment and monitoring of lateonset hypogonadism in males: ISA, ISSAM, EAU, EAA and ASA recommendations. Eur J Endocrinol.

[23] Bhasin S, Cunningham GR, Hayes FJ, Matsumoto AM, Synder PJ, Swerdloff RS (2006). Testosterone therapy in adult men with androgen deficiency syndromes; an Endocrine Society Clinical Practice guideline. J Clin Endocrinol Metab.

[24] Ramasamy R, Armstrong JM, Lipschultz LI (2015). Preserving fertility in the hypogonadal patient: an update. Asian J Androl.

[25] Anand-Ivell R, Wohlgemuth J, Haren MT, Hope PJ, Hatzinikolas G, Wittert G (2006). Peripheral INSL3 concentrations decline with age in a large population of Australian men. Int J Androl.

[26] Cuban S, Doluogu OG, Keles I, Demirci H, Turkoglu AR, Guzelsoy M (2016). Age and total and free prostate-specific antigen levels for predicting prostate volume in patients with benign prostatic hyperplasia. The Aging Male.

[27] Kayikci A, Cam K, Kacagan C, Tekin A, Ankarali H (2012). Free prostate-specific antigen is a better tool than total prostate-specific antigen at predicting prostate volume in patients with lower urinary tract symptoms. Urology.

